# The Predictive Role of the Triglyceride/High-Density Lipoprotein Ratio and the Triglyceride–Glucose Index, Along with Anthropometric Measurements, in Diagnosing Non-Alcoholic Fatty Liver Disease in Obese Kids and Juveniles, and the Evaluation of Novel Cardiovascular Risk Markers in Pediatric NAFLD

**DOI:** 10.3390/children12111439

**Published:** 2025-10-24

**Authors:** Emrah Çığrı, Funda Çatan İnan, Sedat Gülten, Mehmet Akif Bildirici, Ayşe Ece Gökkaya, Metin Asıleren, Mustafa Koyun, Bahadır Reis, Merve Esen

**Affiliations:** 1 Department of Paediatrics, Faculty of Medicine, Kastamonu University, 37150 Kastamonu, Türkiye; ayseecegokkaya@gmail.com (A.E.G.);; 2 Department of Biostatistics and Medical Informatics, Bilecik Seyh Edebali University, 11230 Bilecik, Türkiye; 3 Department of Biochemistry, Faculty of Medicine, Kastamonu University, 37150 Kastamonu, Türkiye; 4 Department of Radiological, Faculty of Medicine, Kastamonu University, 37150 Kastamonu, Türkiye; 5 Nutrition and Dietetics, Kastamonu Training and Research Hospital, Kastamonu University, 37150 Kastamonu, Türkiye

**Keywords:** children, obesity, lipid metabolism

## Abstract

**Highlights:**

Our article is about practical methods that we can use in the early detection of non-alcoholic fatty liver disease, which is common in obese children.

**What are the main findings?**

- TG/HDL ratio and TyG index show a significant increase in obese children with NAFLD.
- Cardiovascular risk indices increase in obese children with NAFLD.

**What is the implication of the main finding?**

- TG/HDL ratio and TyG index are very significant parameters in the early detection of NAFLD.
- The development of NAFLD in obese children also increases the risk of cardiovascular disease.

**Abstract:**

Aim: This current research aims to determine the predictive value of the ratio of triglyceride (TG)/high-density lipoprotein cholesterol (HDL-C), index of triglyceride–glucose (TyG), homeostatic model assessment for insulin resistance (HOMA-IR) score, and anthropometric measurements at the onset of non-alcoholic fatty liver disease (NAFLD) in obese kids and juveniles. It also sought to assess how novel cardiovascular risk markers are affected in obese pediatric patients with NAFLD. Materials and Methods: Between November 2024 and May 2025, a total of 199 pediatric patients were prospectively evaluated, including 150 children with obesity and 49 entirely healthy controls. Two categories of obese patients were created based on whether or not they had non-alcoholic fatty liver disease. These groups were compared with each other and with the control group in terms of HOMA-IR score, index of TyG, ratio of TG/HDL-C, anthropometric parameters (percentage of body fat [BFP], index of body mass [BMI], body fat mass [BFM], waist circumference [WC]), and cardiovascular risk markers. The cutoff values, sensitivity, and specificity of the HOMA-IR score, ratio of TG/HDL-C, anthropometric measurements, and index of TyG in predicting NAFLD were assessed using Receiver Operating Characteristic (ROC) analysis. Results: Obese kids and juveniles with NAFLD had significantly higher TG/HDL-C ratios, TyG indices, HOMA-IR scores, anthropometric measurements, and cardiovascular risk markers than those without NAFLD (*p* < 0.001). The TG/HDL-C ratio (AUC: 0.936; 81.8% sensitivity, 95.9% specificity) and the TyG index (AUC: 0.912; 81.8% sensitivity, 91.8% specificity) showed strong predictive value for NAFLD, while HOMA-IR and WC were found to be relatively weaker predictors. Conclusions: The index of TyG and ratio of TG/HDL-C are highly effective parameters in predicting NAFLD development in obese kids and juveniles. Those with increased WC and BFP should be closely monitored for NAFLD development. Pediatric patients with NAFLD should be carefully followed up for potential cardiovascular diseases.

## 1. Introduction

Chronic liver illness, known as non-alcoholic fatty liver disease (NAFLD), is typified by excessive hepatic fat deposition, usually coupled with high hepatic enzyme levels [1,2]. NAFLD is linked to cardiometabolic risk elements, including insulin resistance, dyslipidemia, and obesity, and is thought to be a major reason for end-stage liver disease and liver fibrosis [3].

Over the past ten years, the existence of non-alcoholic fatty liver disease (NAFLD) in kids and juveniles has risen by 62%. Almost 1/3 of obese boys and 1/4 of obese girls have NAFLD [4,5].

NAFLD has been demonstrated to indicate an elevated risk of cardiovascular disease and is strongly linked to obesity, dyslipidemia, and type 2 diabetes mellitus [6,7]. Therefore, Public health benefits from early detection of NAFLD, and a straightforward, efficient diagnostic tool could be beneficial for identifying and managing affected individuals. This study aims to investigate simple, non-invasive methods for early detection of NAFLD in obese kids and juveniles and to evaluate how novel cardiovascular risk markers are affected in this population.

The index of triglyceride–glucose (TyG), determined utilizing the amount of fasting glucose and TG, is a low-cost and basic surrogate indicator for IR [8]. Studies in adults have shown that the TG/HDL-C proportion is a reliable indicator of IR and metabolic syndrome [9]. These markers are advantageous due to their non-invasive and easy-to-use nature. However, there are limited studies examining their effectiveness in children with NAFLD.

## 2. Materials and Methods

This investigation included 199 children and adolescents in total who presented to the Pediatric Clinic of Kastamonu Training and Research Hospital from November 2024 to May 2025, comprising 150 obese and 49 healthy individuals. Obesity was described as a BMI (weight/height^2^ in kg/m^2^) ≥ 95th percentile. Obese participants were split into 2 groups: those diagnosed with NAFLD based on higher aspartate aminotransferase (AST), alanine aminotransferase (ALT), or total abdominal ultrasound (Group 1, *n* = 77), and those without NAFLD (Group 2, *n* = 73). According to NASPGHAN standards, ALT is the most effective screening test for children’s NAFLD. According to these recommendations, ALT should be at least 80 U/L at the first screening or double the customary upper limit during a follow-up (ALT should be at least 44 U/L for girls and 52 U/L for boys) [10]. In our investigation, the diagnosis of NAFLD was made based on ALT elevation, and the decision was made according to NASPGHAN recommendations. A control group was formed with age- and sex-matched, entirely healthy children and adolescents with no obesity or chronic illness (Group 3, *n* = 49).

Health status in the control group was confirmed by:

∗ Reviewing hospital records to determine any history of chronic illness
∗ Taking a detailed medical history from the family
∗ Performing comprehensive blood tests
∗ Assessing growth and development parameters and vital signs

Measurements included glucose, insulin, TG, low-density lipoprotein cholesterol (LDL-C), total cholesterol (TC), and HDL-C. Non-HDL-C was calculated using the formula: TC-HDL-C [11], HOMA-IR was determined using the rubric: (glucose × insulin)/405, and scores > 3.42 were regarded as HOMA-IR (+) [12]. The TG/HDL-C ratio, TyG index [log(TG × glucose/2)] [13], atherogenic index of plasma (AIP = log[TG/HDL]) [14], atherogenic coefficient (AC = non-HDL-C/HDL-C) [15], Castelli Risk Index-I (CRI-I = TC/HDL-C), and Castelli Risk Index-II (CRI-II = LDL-C/HDL-C) [16] were also calculated.

At the midway point between the iliac crest top and the lower edge of the final perceptible rib at the expiration end, the WC was measured using a measuring tape. BFM and body fat percentage (BFP) were determined using a TANITA MC-780S/ST Segmental Body Composition Analyzer (Daisen, Akita, Japan) for children. The measurements were carried out by a single professional in this field. After ten minutes of rest, systolic and diastolic blood pressure readings were taken using an age-appropriate cuff on the right upper arm while seated.

The 3 groups were compared on the basis of demographic information, TG/HDL-C ratio, index of TyG, AC, AIP, CRI-I, and CRI-II. Additionally, Group 1 and Group 2 were compared based on HOMA-IR score, BMI, BFP, BFM, WC, and diastolic and systolic blood pressure.

Following ten to twelve hours of fasting, all blood samples were obtained after obtaining informed consent. Glucose, TG, TC, HDL-C, and LDL-C serum concentrations were measured with spectrophotometry (Beckman Coulter AU 5800, CA, USA), while insulin concentrations were measured using chemiluminescence technology (Beckman Coulter Unicel DXI 800, CA, USA).

### 2.1. Exclusion Criteria

The research did not include individuals with syndromic or genetic obesity, obesity because of endocrine dysfunction (e.g., hypothyroidism, celiac disease, Cushing Syndrome), long-term use of sulfonylureas, steroids, tricyclic antidepressants, or antihypertensive medications, or those with previously diagnosed diabetes.

### 2.2. Statistical Analysis

For all analyses, SPSS 26 software was used. Normality analysis was tested by skewness and kurtosis. A chi-square test for categorical variables and a one-way ANOVA for continuous variables were employed to evaluate the Clinical characteristics of the NAFLD participants, non-NAFLD participants, and control groups. An independent samples t-test was employed to analyze the participants’ characteristics regarding the existence of NAFLD. NAFLD predictability between TG/HDL, TyG, BFP, WC, and HOMA-IR was compared using analysis of the area under the ROC curve (AUC) and Receiver Operating Characteristic (ROC). After calculating the variables’ sensitivity, specificity, and Youden index, the point with the highest Youden index was applied to figure out the ideal cut-off value for identifying NAFLD. A 2-tailed P-value of less than 0.05 was taken to be significant.

Ethical Approval: Kastamonu University granted approval for this study through the Non-Interventional Clinical Research Ethics Committee on 23 October 2024, with decision number 2024-KAEK-90.

## 3. Results

The investigation analyzed a total of 199 individual participants. Among those with NAFLD, 59.7% had grade 1, 28.5% had grade 2, and 11.8% had grade 3 disease. Table 1 presents a comparison of the three groups with regard to demographic data, lipid parameters, and cardiovascular risk indices. The demographic features of the three groups did not differ statistically significantly (*p* > 0.05) (Table 1). However, when it came to lipid metrics, there were notable variations between the groups. Grp1 had a significantly greater ratio of TG/HDL-C and index of TyG than Grps 2 and 3 (*p* < 0.001) (Table 1).

Table 2 shows the comparison between Grp1 and Grp2 according to HOMA-IR score, blood pressure, and anthropometric scores.

**Figure 1:**
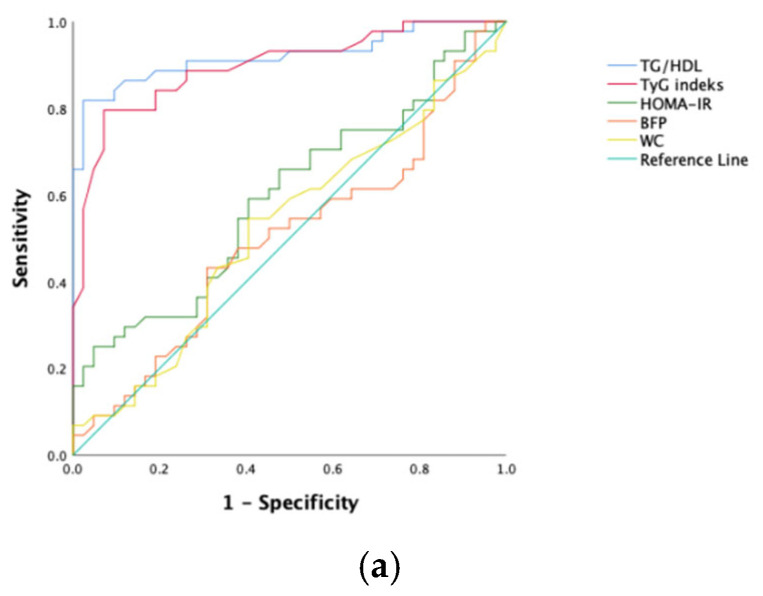

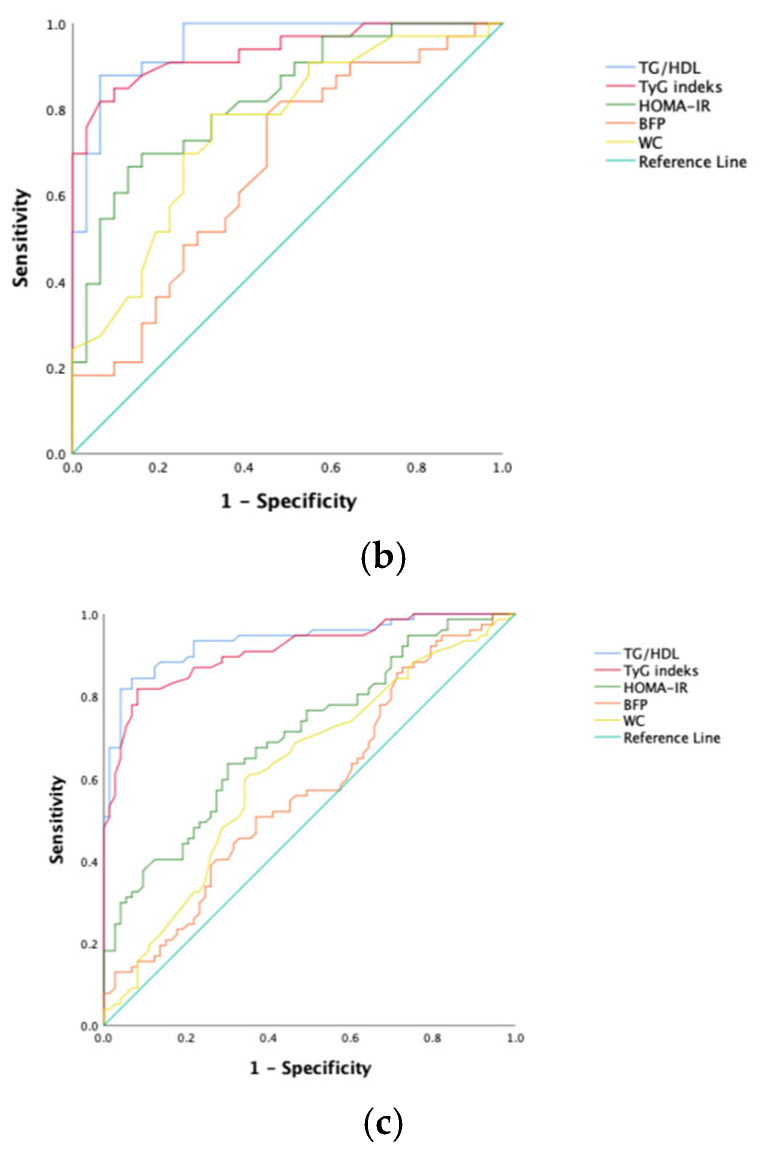
ROC curves of each NAFLD predicting parameter. (**a**) Female; (**b**) male; (**c**) all patients.

To predict NAFLD development in obese kids and juveniles, the sensitivity, specificity, and optimal cutoff values for the index of TyG, ratio of TG/HDL-C, score of HOMA-IR, BFP, and WC were calculated via ROC analysis (Table 3). The ROC curves are shown in Figure 1. Among all participants, the ratio of TG/HDL-C and the index of TyG had the greatest predictive power (AUC values, respectively, of 0.936 and 0.912). However, HOMA-IR and waist circumference had poor AUC values.

## 4. Discussion

Cardiovascular diseases are the main cause of morbidity and mortality in adults, and atherosclerosis—an underlying factor—often begins in childhood [17]. Therefore, simple and effective tools to assess cardiovascular risk during childhood are urgently needed. While metabolic syndrome is considered a strong predictor of cardiovascular disease, its components are difficult to unify, and no consensus exists on its diagnostic criteria. Consequently, the TG/HDL-C proportion has recently gained attention as a potential surrogate indicator. According to Kinga et al. [18], the ratio of TG/HDL-C was identified as a promising indicator for predicting NAFLD in obese kids and juveniles. Similarly, Adeola et al. [19] argued that the likelihood of having a high ratio of TG/HDL-C was 3.4 times greater in individuals with metabolic syndrome when compared to those without, and they suggested its potential as a basic and rapid screening tool for pediatric metabolic syndrome. In another study by Hyun [20], the ratio of TG/HDL-C was significantly greater in obese kids with severe NAFLD than in those with mild to moderate NAFLD, suggesting it as an effective diagnostic tool for severe NAFLD. A large-scale Korean cohort study of 2721 adolescents aged 10–18 years [21] showed a meaningful association between the TG/HDL-C proportion and metabolic syndrome and concluded that this proportion was a more robust predictor than HOMA-IR. In alignment with the existing literature, our study also proved that obese kids and juveniles with NAFLD had significantly greater TG/HDL-C ratios than their counterparts without NAFLD and healthy controls. We concluded that the TG/HDL-C proportion is a highly effective tool to predict NAFLD among obese youth.

Hepatocellular triglyceride accumulation is a hallmark of NAFLD [22]. Although lipid storage is not a primary function of the liver, hepatic fat accumulation can result from excessive fatty acid intake, carbohydrate-derived synthesis, or hepatic de novo lipogenesis. Hence, an imbalance in lipid and carbohydrate metabolism is central to NAFLD pathogenesis. Additionally, insulin resistance (IR) caused by lipid dysregulation reduces glucose uptake in muscles, enhances glycolysis, and enhances the release of free fatty acids from adipose tissue, ultimately promoting hepatic uptake of fatty acids and triglycerides [23]. The TyG index underscores the close link between IR and NAFLD. Insulin promotes adipocyte differentiation and fatty acid uptake from circulating lipoproteins, while hypertriglyceridemia enhances FFA delivery to the liver and hepatic glucose production, leading to organ steatosis [24]. In our study, Obese kids with NAFLD had substantially greater HOMA-IR scores and TyG indices than kids without the disease. Notably, the index of TyG proved to be a particularly effective diagnostic marker for predicting NAFLD. A systematic review of eight cross-sectional studies conducted in five different countries involving patients aged 2–20 years also concluded that the TyG index is a reliable predictor of IR and other cardiometabolic risk factors in pediatric populations [25]. Kinga et al. [18] also reported the index TyG as a powerful predictor of NAFLD in obese children and adolescents. Guo et al. [26] found a positive correlation among the index TyG and both hepatic fibrosis and steatosis severity in NAFLD. Similarly, Zhang et al. [2] concluded that the index of TyG outperformed ALT testing in predicting NAFLD. Other studies [27,28] also support the positive correlation between the index of TyG and NAFLD in obese pediatric individuals.

Visceral abdominal adiposity leads to increased circulating FFAs, a key driver of NAFLD development [29]. Studies [30,31] have shown that central adiposity is closely linked with cardiometabolic risk in obese kids and juveniles and that indices of central fat accumulation, such as WC, should complement BMI during screening for metabolic risk. In an investigation by Choi et al. [32] on juveniles having NAFLD, most subjects had elevated BMI and BFP, leading the authors to recommend using both metrics to reflect body fat and determine those who are susceptible to disorders like NAFLD linked to obesity. Williams et al. [33] also reported that a BFP over 25% in boys and 30% in girls constitutes a substantial risk contributor to heart disease and NAFLD. Consistent with these conclusions, our research also revealed noticeably greater BMI, BFP, and WC in obese kids and juveniles with NAFLD compared to those without. But we also found that BFP and WC alone were relatively weak predictors of NAFLD in this population.

The Atherogenic Index of Plasma (AIP) is an emerging indicator that reflects complex interactions among various lipoproteins and correlates well with cardiovascular risk [34]. Prior studies have found a significant association between obesity and AIP [35], and AIP has been shown to be a strong predictor in conditions such as acute coronary syndrome, a major cause of cardiovascular mortality [36].

The Atherogenic Coefficient (AC) is another recently proposed cardiovascular risk marker. Defined as the proportion of cholesterol non-HDL to cholesterol HDL, it reflects the burden of atherogenic LDL particles within total cholesterol. Higher AC values indicate increased cardiovascular disease risk [37]. In another investigation by Sujatha et al. [38], elevated AC values were significantly associated with stroke occurrence.

Castelli Risk Indices I and II (CRI-I and CRI-II) are important cardiovascular risk indicators with greater predictive power than isolated lipid parameters [39]. These indices have been associated with stroke risk in both males and females [37,38].

Viktoriya et al. [40] reported substantially greater values of AIP, AC, CRI-I, and CRI-II in obese kids with metabolic syndrome compared to healthy obese children and controls. Similarly, Song et al. [24] found elevated values of these indices in obese kids having NAFLD than in those kids without the disease. Our findings are consistent with the literature: all four cardiovascular risk indices—AIP, AC, CRI-I, and CRI-II—were significantly higher in obese children with NAFLD compared to those without and to the control group. These results indicate that children with NAFLD have significantly worse lipid profiles and higher cardiovascular risk markers than both their non-NAFLD peers and healthy controls.

In obese kids and juveniles, increases in the TG/HDL-C ratio, TyG index, HOMA-IR score, and anthropometric measurements during routine follow-ups should raise suspicion about the onset of NAFLD. Kids diagnosed with NAFLD should also be closely monitored for cardiovascular risk during follow-up evaluations.

## 5. Limitations

One major restriction to our research is the absence of information on pubertal status. Since HOMA-IR values tend to increase with puberty, the absence of this information may have influenced our findings. Another limitation of our study is that using ALT, AST elevation, and ultrasonography as NAFLD diagnostic criteria may miss mild cases.

## Figures and Tables

**Table 1:** Comparison of demographic data, lipid parameters, and cardiovascular risk indices among the three groups.

	Group 1 NAFLD *n* = 77 (38.7%)	Group 2 Non-NAFLD *n* = 73 (36.7%)	Group 3 Control *n* = 49 (24.6%)	Test-Value	*p*	Post Hoc
Age	11.81 ± 2.90	11.75 ± 2.95	11.73 ± 2.73	0.011	0.989	
Gender						
Female	44 (57.1%)	42 (57.5%)	28 (57.1%)	0.003	0.999	
Male	33 (42.9%)	31 (42.5%)	21 (42.9%)			
TG/HDL	3.91 ± 1.96	1.57 ± 0.56	1.11 ± 0.47	92.335	<0.001	1−2, 1−3, 2−3
TyG index	3.81 ± 0.18	3.5 ± 0.14	3.37 ± 0.14	124.04	<0.001	1−2, 1−3, 2−3
AIP	0.54 ± 0.20	0.16 ± 0.17	0.01 ± 0.18	142.38	<0.001	1−2, 1−3, 2−3
AC	2.86 ± 0.77	2.15 ± 0.52	1.73 ± 0.45	54.85	<0.001	1−2, 1−3, 2−3
CRI-I	3.86 ± 0.77	3.15 ± 0.52	2.73 ± 0.45	54.85	<0.001	1−2, 1−3, 2−3
CRI-II	2.57 ± 0.61	1.95 ± 0.41	1.58 ± 0.35	66.42	<0.001	1−2, 1−3, 2−3

TG/HDL: Triglyceride/High-density lipoprotein cholesterol, TyG index: Triglyceride-glucose index, AIP: Plasma atherogenic index, AC: Atherogenic coefficient, CRI-I: Castelli risk index-I, CRI-II: Castelli risk index-II.

**Table 2:** Comparison of HOMA-IR score, blood pressure, and anthropometric measurements between Group 1 and Group 2.

	Group 1 NAFLD *n* = 77 (51.3%)	Group 2 Non-NAFLD *n* = 73 (48.7%)	Test-Value	*p*
HOMA-IR	4.24 ± 2.43	2.66 ± 1.30	5.008	<0.001
BMI (kg/m^2^)	28.61 ± 5.12	26.34 ± 4.43	2.879	0.005
BFP (%)	36.69 ± 8.42	33.85 ± 6.73	2.272	0.025
BFM (kg)	25.72 ± 11.93	20.61 ± 9.66	2.871	0.005
WC (cm)	101.22 ± 13.92	95.27 ± 13.73	2.632	0.009
Systolic (mmHg)	122.69 ± 11.89	117.19 ± 12.59	2.749	0.007
Diastolic (mmHg)	68.78 ± 10.11	66.85 ± 7.78	1.313	0.191

HOMA-IR: Insulin Resistance Homeostatic model, BMI: Body Mass Index, BFP: Body Fat Person, BFM: Body Fat Mass, WC: Waist Circumference.

**Table 3:** Cutoff values and areas under the ROC curves for each variable for predicting NAFLD.

Variables	Cutoff	Sensitivity	Specificity	Youden Index	AUC (95% CI)	*p*
**All subject**						
TG/HDL	2.40	0.818	0.959	0.777	0.936	<0.001
TyG	3.66	0.818	0.918	0.736	0.912	<0.001
HOMA-IR	3.09	0.636	0.699	0.335	0.705	<0.001
BFP	30.25	0.857	0.288	0.145	0.574	0.118
WC	99.5	0.597	0.658	0.255	0.621	0.011
**Female**						
TG/HDL	2.46	0.818	0.976	0.794	0.921	<0.001
TyG	3.66	0.795	0.929	0.724	0.897	<0.001
HOMA-IR	5.01	0.25	0.952	0.202	0.596	0.124
BFP	31.75	0.432	0.690	0.122	0.501	0.993
WC	99.5	0.545	0.595	0.140	0.524	0.704
**Male**						
TG/HDL	2.31	0.879	0.935	0.814	0.954	<0.001
TyG	3.72	0.818	0.935	0.753	0.935	<0.001
HOMA-IR	3.35	0.667	0.871	0.538	0.825	<0.001
BFP	35.30	0.485	0.742	0.227	0.669	0.020
WC	93.5	0.788	0.677	0.465	0.756	<0.001

TG/HDL: Triglycerides/High-density lipoprotein cholesterol, TyG index: Triglyceride-glucose index, HOMA-IR: Insulin Resistance Homeostatic model, BFP: Body Fat Percentage, WC: Waist Circumference.

## Data Availability

The original data presented in the study are openly available at https://docs.google.com/spreadsheets/d/1dfcqMuKgaNgGqyoafnoYlEwTBhFZZj7v/edit?gid=1530977858#gid=1530977858 (accessed on 26 August 2025).
